# Anoxia Rapidly Induces Changes in Expression of a Large and Diverse Set of Genes in Endothelial Cells

**DOI:** 10.3390/ijms24065157

**Published:** 2023-03-08

**Authors:** Antonella Antonelli, Emanuele Salvatore Scarpa, Santina Bruzzone, Cecilia Astigiano, Francesco Piacente, Michela Bruschi, Alessandra Fraternale, Christian A. Di Buduo, Alessandra Balduini, Mauro Magnani

**Affiliations:** 1 Department of Biomolecular Sciences, University of Urbino Carlo Bo, 61029 Urbino, Italy; 2 Department of Experimental Medicine, Section of Biochemistry, Viale Benedetto XV 1, 16132 Genova, Italy; 3 Department of Molecular Medicine, University of Pavia, 27100 Pavia, Italy; 4 Department of Biomedical Engineering, Tufts University in Boston, Boston, MA 02111, USA

**Keywords:** anoxia, endothelial cells, interleukin/cytokine expression, transcriptome analysis, SIRT6

## Abstract

Sinusoidal endothelial cells are the predominant vascular surface of the bone marrow and constitute the functional hematopoietic niche where hematopoietic stem and progenitor cells receive cues for self-renewal, survival, and differentiation. In the bone marrow hematopoietic niche, the oxygen tension is usually very low, and this condition affects stem and progenitor cell proliferation and differentiation and other important functions of this region. Here, we have investigated in vitro the response of endothelial cells to a marked decrease in O_2_ partial pressure to understand how the basal gene expression of some relevant biological factors (i.e., chemokines and interleukins) that are fundamental for the intercellular communication could change in anoxic conditions. Interestingly, mRNA levels of *CXCL3*, *CXCL5*, and *IL-34* genes are upregulated after anoxia exposure but become downmodulated by sirtuin 6 (SIRT6) overexpression. Indeed, the expression levels of some other genes (such as *Leukemia Inhibitory Factor* (*LIF*)) that were not significantly affected by 8 h anoxia exposure become upregulated in the presence of SIRT6. Therefore, SIRT6 mediates also the endothelial cellular response through the modulation of selected genes in an extreme hypoxic condition.

## 1. Introduction

In the bone marrow (BM) hematopoietic niche, the oxygen tension is usually very low, and this condition affects stem and progenitor cell proliferation and differentiation. The partial pressure of oxygen (pO_2_) at a cellular level regulates hematopoietic growth factors, chemokines, and adhesion molecule expression that, in turn, affect the proliferation and maturation of other cellular components of the niche. Sinusoidal endothelial cells are the predominant vascular surface of the BM and constitute the functional hematopoietic niche. These cells comprise the platform where trafficking into and out of the BM occurs and where hematopoietic stem and progenitor cells harbor and receive cues for self-renewal, survival, and differentiation. Several authors have contributed in defining niches and mobilization pathways for hematopoietic stem and progenitor cells; this includes the identification of some of the cell types involved, such as osteoblasts, adventitial reticular cells, endothelial cells, monocytic cells, and granulocytic cells, as well as the main factors that anchor stem cells in the niche and/or induce their quiescence, such as vascular cell adhesion molecule (VCAM)-1, CD44, hematopoietic growth factors (e.g., stem cell factor (SCF)), and chemokines, including IL-12 and IL-8 [1]. While elucidating the basic mechanisms of intracellular cross-talk between the different cell components of the vascular niche, and considering the role of oxygen in the hematopoietic niche, we recently demonstrated, through an in vitro anoxic model using monocyte–macrophage-type cells representative of components of the hematopoietic niche, that modulation of pO_2_ has an effect on the modulation of the expression of some cytokines [2]. Reduced oxygen tension has been shown to enhance the production of erythroid, megakaryocytic, and granulocytic–monocytic progenitors in vitro. Due to the inaccessibility of the bone marrow to direct noninvasive oxygen measurements, some authors have used mathematical modeling of pO_2_ distributions in the bone marrow and speculated that stem cells are located at regions with very low pO_2_ levels (almost anoxic) because this prevents oxygen radicals from damaging these important cells [3]. The modulating effects of vascular endothelial growth factor (VEGF) are essentially limited to endothelial cells, the only cell type consistently shown to express VEGF receptors. It has already been demonstrated that the induction of *VEGF* mRNA in Human Umbilical Vein Endothelial Cells (HUVECs) was dependent on the degree of hypoxic stimulation [4,5].

Here, we report data collected during studies performed using an in vitro cell model of anoxia. HUVECs were chosen as a cell type that can mimic endothelial cells present around the sinusoidal vessels of the vascular hematopoietic niche that live at low O_2_ conditions. We investigated in vitro how an extreme reduction in O_2_ percentage of the HUVEC environment can alter the basal expression of some genes. Moreover, we determined the levels of chemokines, interleukins, and other molecules that are affected by pO_2_ changes in the culture medium. We started by investigating the cellular response to anoxic conditions following *Vascular Endothelial Growth Factor (VEGF*) gene expression given its role as a key regulator of angiogenesis and hematopoiesis [6,7]. The first studies determined the length of anoxic exposure required to obtain maximum levels of *VEGFA* mRNA expression (8 h of anoxia) in our HUVEC model; all subsequent scientific evaluations here reported were performed using this time point. As it is known that *VEGFA* is one of the genes upregulated by HIF-1α functionally induced in low-oxygen conditions, we analyzed *HIF1A* mRNA and HIF-1α protein levels in cell extracts of HUVECs after 8 h of anoxia. Under normoxic conditions, the α subunit of HIF-1 is hydroxylated by prolyl hydroxylases (PHDs), which causes it to be recognized by the protein product of the von-Hippel-Lindau (VHL) gene, ubiquitinated, and degraded by the proteasome [8]. When cells are at a low O_2_ concentration, the PHDs are not active; consequently, HIF-1α is not degraded but can translocate to the nucleus, where it can dimerize with the β subunit. The heterodimeric transcription factor induces the transcription of genes mediating cellular adaptation to a low oxygen environment. Moreover, it is known that SIRT6, a member of the NAD-dependent deac(et)ylases sirtuin family, is involved in the regulation of several different biological processes, including metabolism, DNA repair, and aging and can regulate HIF-1α in different ways. For example, it has been demonstrated that SIRT6 functions as a co-repressor of HIF-1α transcriptional activity, with metabolic effects such as inhibition of glucose uptake and glycolysis [9]. Recent research has also shown how SIRT6 under oxidative stress prevents the degradation of HIF-1α by deubiquitination at two specific K residues, thereby promoting angiogenesis [10]. Overall, the aim of this study was to investigate how a marked decrease in O_2_ affects gene expression in HUVECs, with a focus on the production of molecules such as chemokines and interleukins and on the ability of SIRT6 to modulate the response when endothelial cells are found at extremely low O_2_ levels.

## 2. Results and Discussion

In our experimental cell model, we have taken the hypoxic condition to the extreme. By flushing an appropriate 95% N_2_ and 5% CO_2_ gas mixture in the Hypoxia Incubator Chamber, endothelial cells were exposed to very low pO_2_ levels (almost 0–0.5% oxygen or anoxia). Some authors have hypothesized that regions with very low pO_2_ levels (almost anoxic) exist in the bone marrow [11] where they prevent oxygen radicals from damaging stem cells [3]. We used an anoxic cell model to investigate how sinusoidal vessel endothelial cells living in the vascular hematopoietic niche could also be affected by very low pO_2_ levels leading to the up- or downregulation of specific gene targets.

**Figure 1:**
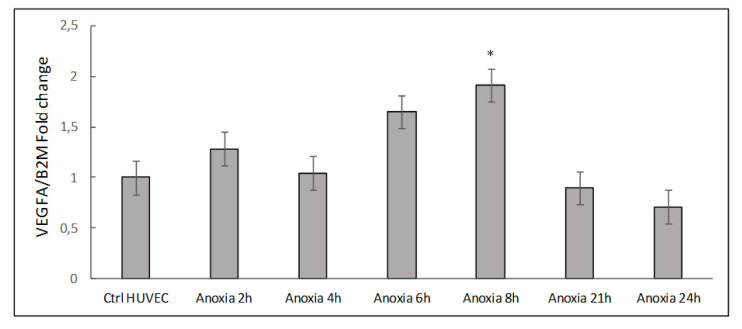
Analysis of mRNA levels of *VEGFA* in cell extracts of HUVECs exposed to anoxia stimulus from 2 to 24 h. Values are expressed as mean ± SD; *n* = 2, * p < 0.05 compared with normoxic cells.

To test for anoxia-specific regulation, we followed *VEGFA* gene expression as a biomarker. The mRNA levels of *VEGFA* were analyzed by RT-q-PCR in cell extracts of HUVECs maintained for different times (2, 4, 6, 8, 21 and 24 h) under anoxic conditions obtained by using the Hypoxic incubator Chamber as reported in the Materials and Methods. Cells maintained in normoxia (21% O_2_) were used as control samples. Figure 1 shows a representative time-course experiment performed with the HUVEC cell model exposed to anoxia. The results indicate an upregulation effect on *VEGF* gene expression, whose mRNA levels were already increased after 2 h of treatment (1.28 ± 0.29 fold), reaching a maximum peak of expression from 6 to 8 h (1.92 ± 0.28 fold, *p* = 0.02) and returning to basal levels after 24 h. To validate the time-point showing the highest peak of *VEGFA* expression, we performed additional experiments to evaluate *VEGFA* and *HIF1A* mRNA levels at 8 h under anoxic conditions.

**Figure 2:**
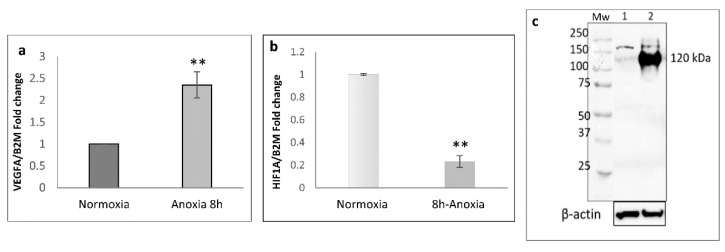
Analysis of mRNA levels of *VEGFA* (**a**) and *HIF1A* (**b**). Values are expressed as mean ± SD; *n* = 2, ** p < 0.01 when compared with normoxic cells. (**c**) Immunoblotting analysis of HIF-1α protein in same cell extracts of HUVEC exposed to 8 h anoxia. 20 µg of total extract protein were loaded in each lane (lane 1; normoxia; lane 2; 8 h anoxia). β-actin was detected as protein loading control.

Figure 2a shows the increased *VEGF* mRNA expression reaching a 2.35 ± 0.29-fold change, confirming the data obtained during the time course (Figure 1); interestingly, real-time quantitative PCR analysis performed in parallel in the same cell extracts derived from anoxic and normoxic cell samples to determine the gene expression of *HIF1A* (since hypoxia-inducible factor 1 α is the inducible subunit of the HIF-1 transcription factor) showed a decrease of *HIF1A* mRNA levels (0.23 ± 0.05-fold) in cell extracts of 8 h anoxic HUVEC samples with respect to control cells (Figure 2b).

Moreover, HIF-1α protein was detected by western immunoblotting analysis; as expected, HIF-1α was accumulated in cell cytoplasm upon HUVEC challenged with anoxia (Figure 2c).

Data support the involvement of this important transcriptional effector regulating the responses of HUVECs exposed to anoxic stimulus, evidencing an accumulation of a 120 kDa protein band in 8 h anoxia-treated samples in comparison with cells maintained for the same time in normoxic conditions. These results are in agreement with the literature; when oxygen is not present in adequate amounts, hydroxylation cannot occur, which means that HIF1-α can be stably expressed [12]. As a result, this protein can bind to HIF1β and facilitate gene transcription of many HIF target genes. Despite this increased amount of HIF 1α protein being clearly evident after an 8 h anoxia treatment, the levels of *HIF1A* mRNA do not appear to be upregulated; on the contrary, in comparison with basal levels of expression, a downregulation occurs. It is commonly accepted that under low oxygen (hypoxic) conditions, the hydroxylase activity of the PHD enzymes is inhibited; HIF therefore escapes hydroxylation and degradation to initiate a transcriptional program of cellular response and adaptation to hypoxia. However, it was reported in the literature that in some cases, negative regulatory feedback occurs [13]. Moreover, some other authors had already reported that the inhibition of *HIF1A* transcription is necessary to counteract the transcription-dependent degradation of HIF-1α which occurs during hypoxic/anoxic conditions. Under normoxia, HIF1-α is expressed at low levels and is degraded via the classic PHD/VHL pathway and therefore does not activate the feedback loop. Instead, under anoxia/hypoxia, HIF1-α accumulates and transcriptionally activates its own degradation that is independent from the PHD/VHL pathway. Thus, our data, indicating that in our cell model, *HIF1A* mRNA expression levels significantly decrease in 8 h anoxia-treated HUVECs (Figure 2b), further promoting HIF1-α accumulation in HUVEC cells (Figure 2c), are in line with previous reports [14].

### 2.1. Transcriptional Analysis and Evaluation of Chemokine Release

**Figure 3:**
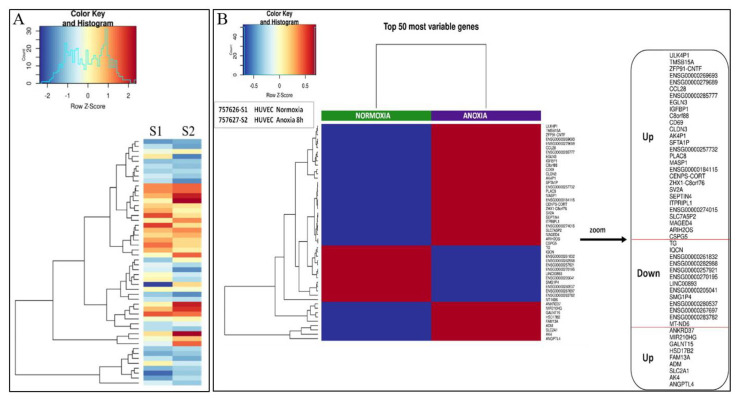
RNA−seq analysis of mRNA expression in HUVEC cells maintained in normoxia (**S1**) or anoxia (**S2**). (**A**) Heatmap generated from transcriptome analysis of **S1** vs. **S2**. (**B**) List of the 50 most variable genes involved in cellular responses differentially expressed in anoxic (**S2**) versus normoxic (**S1**) samples. Red color means upregulated genes; blue color means downregulated genes.

**Figure 4:**
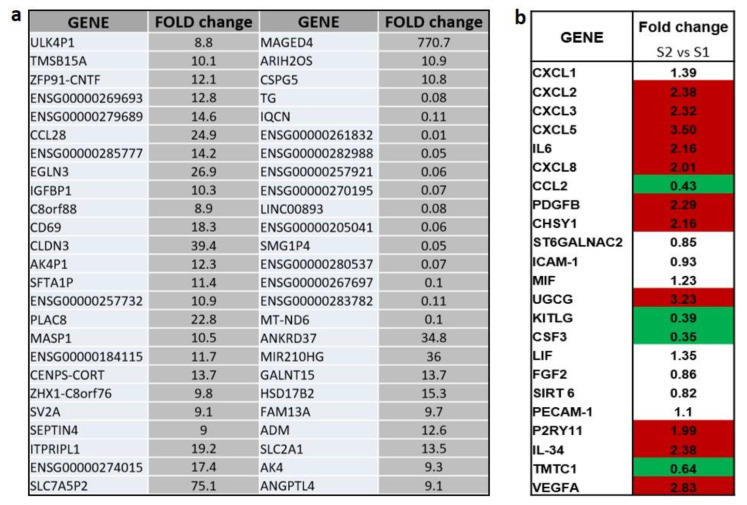
(**a**) Using 2^log FC^ and DEG table provided from BMR Genomics, fold-changes in gene expression of the 50 most variable genes were calculated. (**b**) Increased fold-changes of some upregulated genes (**red color**) in HUVECs maintained for 8 h in anoxic condition (**S2**) with respect to cells in normoxia (**S1**) and decreased fold-changes of some downregulated genes (**green color**) which are said to be involved in the hematopoietic niche environment.

RNA-Seq analysis provided us the top 50 most variable genes found in the total RNA extracted from HUVECs treated or not in anoxia for 8 h as reported in Material and Methods section. A representation of a generated heat map evidencing up- or downregulated genes in anoxic HUVEC sample (S2) respect to normoxic HUVEC sample (S1) was reported in Figure 3. Fold changes in gene expression of the 50 most variable genes screened (Figure 4) are expressed as anoxic (S2) versus normoxic (S1) condition (Figure 4a). In addition to the 50 most variable genes derived from BMR Genomics data, we calculated the fold-changes of those genes that most seemed to be involved in the regulation of the hematopoietic niche environment, such as *VEGFA*, as well as those that mediate the cellular response to anoxia/hypoxia. An increase of at least 1.5-fold and a decrease of at least 0.6-fold in the mRNA levels with respect to basal levels in normoxic condition were considered. The expression of many mediators, such as interleukins including *IL-2, IL-3, IL-5, IL-6, IL-8*, and factors such as *PDGFB* (Platelet Derived Growth Factor Subunit B), *UGCG* (UDP-Glucose Ceramide Glucosyltransferase), *P2RY11* (purinergic receptor), and *VEGFA* (Vascular Endothelial Growth Factor A) were found to be upregulated (>2-fold change), whereas a downregulation of some other genes (<0.5-fold change), such as *CCL2* (C-C Motif Chemokine Ligand 2), *KITLG* (KIT Ligand) and *CSF3* (Colony Stimulating Factor 3 Receptor), was evidenced (Figure 4b).

The secretome profile of anoxia-exposed HUVECs was compared with that of HUVECs kept in normoxic conditions via a BIO-Rad 20-plex detection kit as reported in the Materials and Methods section. The detection of some secreted human interleukins/cytokines was reported in Table 1. VEGF was not considered since it is a supplement of the HUVEC-specific culture medium. The data highlighted a strong increase in protein release, particularly of the inducible protein 10 (IP10) (>50 fold), IL-6 (>7 fold), macrophage chemoattractant protein-1 (MCP-1) (>5 fold), and IL-5 (>2.2 fold), while IL-1b and IL-2 secretion as well as the platelet-derived growth factor (PDGF-BB) appeared to be unaffected by the anoxic environment, with their amounts in the respective cell supernatants being borderline detectable. Since some chemokines were not detected by the BIO-Plex kit, only data of the most reliable factors were reported in Table 1; moreover, a comparison between released proteins and their corresponding mRNA expression was not possible for all the target genes obtained with RNA-Seq reported in Figure 4. For example, the mRNA expression levels for *IL34* and *CXCL3* could not be correlated to the respective protein amounts released in cellular supernatants due to the absence of these factors in the BIO-Plex kit; conversely, for IP-10, the protein amount in the cell supernatant was assessed, but not the mRNA by RNA-Seq.

It is evident that in our experimental cell model, the anoxia stimulus leads to a modulation of mRNA expression of key cytokines having a role in the control of cellular metabolism, energetics, and post-transcriptional gene regulation by O_2_. Thus, understanding the effect of low pO_2_ levels on endothelial cells could be important to identify which biological factors are involved in the anoxic response with the aim of investigating the possible cell activity of sinusoid endothelial cells of hematopoietic tissues living in extreme hypoxia conditions. Significantly increased *CXCL2, CXCL8, CXCL3, CXCL5, PDGFB,* and *VEGFA* mRNA levels (≥2-fold) detected by transcriptional analyses could be connected to the modulation of specific biological pathways in anoxic/hypoxic conditions since oxygen tension (pO_2_) is an important determinant of hematopoietic stem and progenitor cell proliferation and differentiation. For example, IL-3 was one of the earliest cytokines implicated in self-renewal, proliferation, and differentiation of primitive and mature hematopoietic stem cells. Although the role of IL-3 in the bone marrow has not been fully unraveled, it is a cytokine with multilineage potential, and it is known that its action shows functional redundancy with other cytokines such as the granulocyte-macrophage colony-stimulating factor (GM-CSF) and IL-5. IL-3 was shown to increase and decrease the expansion and/or self-renewal capacity of adult HSCs; however, it was also evidenced that IL-3 supports the growth of early hematopoietic progenitors and promotes their response to other, later-acting cytokines [15]. Moreover, IL-3 was reported to be able to induce the expression of interleukin 2 (IL-2) receptor (IL-2R) (CD25) on a subset of early myeloid cells in normal human bone marrow that had been first depleted of mature hematopoietic cells [16]. The role of IL-2 in HSC maintenance is unknown and some studies suggest that equilibrium between IL-2 and IFN-γ is critical for steady state hematopoiesis [17].

Abundant literature reports VEGF as a potent mitogen and permeability factor for endothelial cells playing a central role in angiogenesis, inflammation, and cancer. VEGF also mediates the homeostatic adaptation to hypoxic conditions by promoting an increase in vascular density to compensate for decreased oxygenation; therefore, the synthesis and secretion of VEGF is required for the maintenance of the integrity of the vascular system. Furthermore, CXCL8/IL8, a potent proangiogenic and inflammatory chemokine, upregulates VEGF mRNA and protein levels in endothelial cells by acting on its receptor, CXCR2 (receptor for IL-8), and this results in the autocrine activation of VEGFR2 (VEGF receptor 2) [18,19].

*IL-34* mRNA levels increased by 2.38-fold in anoxic conditions (Figure 4b). Structurally, IL-34 belongs to the short-chain helical hematopoietic cytokine family but shows no apparent consensus structural domains, motifs, or sequence homology with the other 53 cytokines. IL-34 is a novel cytokine that was identified in 2008 in a comprehensive proteomic analysis as a tissue-specific ligand of the CSF-1 receptor (CSF-1R). This cytokine is involved in several processes; it promotes the proliferation, survival, and differentiation of monocytes and macrophages and also plays an important role in innate immunity and in inflammatory processes promoting the release of proinflammatory chemokines [20]. The pro-angiogenic potential of IL-34 was further confirmed experimentally in vitro, where IL-34 was able to recruit endothelial cells to form vascular structures. In the HUVEC endothelial cell line, IL-34 activates several kinases, including PI3K, Src, FAK, and ERK1/2, which importantly contribute to cell differentiation into vascular cords. These effects were mediated by the interaction between IL-34 and glycosaminoglycans at the cellular surface, indicating a CSF-1R-independent mechanism [21]. Additionally, IL-34 can exert additional pro-angiogenic functions via CSF-1R-dependent mechanisms by inducing the secretion of several factors that contribute to angiogenesis, such as IP-10, MCP-1, and IL-8 in PBMCs [22].

The interferon-gamma-induced protein 10 (IP-10), also classified as *CXCL10*, has been described as a chemokine produced by T cells, monocytes, endothelial cells and keratinocytes after stimulation with IFN-γ. In our setting, IP-10 was found to be significantly increased in cell supernatant after 8 h of anoxic stimulus (>50-fold with respect to control cells). Although IP-10 was named as a protein inducible by IFN-γ [23], other agents, including LPS, IL-1α, IL-6, TNF-α, IFN-α, and IFN-β, were also found to highly induce IP-10 expression in vitro in a variety of cells, including endothelial cells. Despite its well-described function of recruiting leukocytes to sites of inflammation, IP-10 also plays a role in the generation and function of effector cells [24]. Some authors have studied the potential molecular mechanism by which hypoxia/ischemia regulates expression of *CXCL10* in endothelial cells, especially in the cardiac microvascular endothelial cells. *CXCL10* secretion and mRNA expression were increased by hypoxia/ischemia treatment in a time-dependent manner [25]. Monocyte chemoattractant protein-1 (MCP-1/CCL2), that was also found to be secreted after 8 h anoxia in cell supernatants (5-fold increased with respect to control samples), is one of the key chemokines that regulates migration and infiltration of monocytes/macrophages. Both CCL2 and its receptor CCR2 have been demonstrated to be induced and involved in various diseases. Migration of monocytes from the blood stream across the vascular endothelium is required for routine immunological surveillance of tissues, as well as in response to inflammation. The effect of MCP-1 on the migration and proliferation of hematopoietic progenitor cells has been examined [26,27]. Some work reported that cardiac stem cells have been shown to play a protective role against hypoxia-induced injury in vivo via an MCP-1-dependent mechanism [28]. Moreover, an increased release of IL-6 (>7-fold versus control sample) was also found in the cell medium after 8 h under anoxic conditions. It was reported that in hypoxic conditions, increased levels of the IL-6 protein induce changes in endothelial permeability. In fact, IL-6 signaling mediates a vast array of effects in the vascular wall, including endothelial activation, vascular permeability, immune cell recruitment, endothelial dysfunction, as well as vascular hypertrophy and fibrosis [29]. IL-6 production can affect both stromal and hematopoietic cells, for example, stimulating megakaryocyte (Mk) growth and maturation in vitro as well as increasing Mk ploidy. Moreover, some authors have recently reported that abnormal levels of IL-6 might interfere with the stability of the bone marrow hematopoietic microenvironment [30,31]. It was already reported that IL-8 is also responsible for the enhanced proliferation and mobilization of HSCs in the bone marrow [32]. In our anoxic cell model, increased IL-8 mRNA and protein levels were found (Figure 4b and Table 1). 

The role of IL-8 as a mediator of angiogenesis and tumor growth has already been described. Increased IL-8 levels in human umbilical cord segments exposed to hypoxia (compared with normoxic controls) were reported. The incubation of human endothelial cells in hypoxia (PO_2_ ~14–18 mmHg) led to the time-dependent release of IL-8 antigens into the conditioned medium; this was accompanied by increased chemotactic activity for some polymorphonuclear leukocytes, which was blocked by antibodies to IL-8 [33,34].

It is clear that the most detectable chemokines and interleukins, released in culture medium from the anoxic cell model, are part of those biological molecules and growth factors which play pivotal roles in bone marrow vascular niche regulation; it is well known that the different cell components together all play an essential role in the maintenance of bone marrow vascular niche function by secreting large quantities of several molecules with hematopoietic activity, including VEGF, IL-6 and IL-8 [35,36].

After 8 h anoxia treatment, we also found increased expression levels of other types of molecules, such as the *P2Y11* receptor (Figure 4b), a member of the purinergic receptor family. The P2Y11 receptor (P2RY11) has a wide distribution in all cell types relevant for cardiovascular pathology: cardiomyocytes, fibroblasts, endothelial and immune cells [37,38].

Some authors have investigated the role of P2Y11R signaling in vascular dysfunction, (where endothelial cell activation was included) reporting that activation of the P2Y11 receptor (P2Y11R) in human dendritic cells, cardiofibroblasts, and cardiomyocytes was protective against hypoxia/reoxygenation lesions. Thus, P2Y11R activation may also protect blood vessels from vascular injury induced from low pO_2_ levels. In particular, the P2Y11 receptor, activated by ATP, has anti-inflammatory actions which could be implicated in endothelial cell protection in cardiovascular diseases [36,37,38,39,40,41].

*UGCG* (UDP-glucose ceramide glucosyltransferase) mRNA levels were also increased (>3-fold change) in HUVECs following anoxia treatment. UGCG is a key enzyme in the sphingolipid metabolism as it generates glucosylceramide, the precursor for all glycosphingolipids, which are essential for proper cell function. Although UGCG has been associated with several cancer-related processes such as maintaining cancer stem cell properties or multidrug resistance induction, the precise mechanisms underlying these processes are unknown [42].

*CXCL5* (C-X-C motif chemokine ligand 5) mRNA levels were also increased 3-fold after anoxic stimulus. It is known that anoxia/hypoxia also affects the CXC chemokine system, which leads to changes in the level of these chemoattractant cytokines in the cancer microenvironment; moreover, CXC chemokines differ in their effect on angiogenesis. CXCL5 elicits this effect by interacting with the cell surface chemokine receptor CXCR2. Moreover, this chemokine stimulates the chemotaxis of neutrophils possessing angiogenic properties; increasing evidence has indicated that CXCL5 is involved in the tumorigenesis of various malignancies [43].

Next, we investigated the expression of some genes involved in the metabolism of glutathione due to its relevant role in the maintenance of cellular redox homeostasis and the related susceptibility of endothelial cells to oxidant injury. A major initiator of endothelial injury is oxidative stress, which results from an imbalanced state of increased reactive oxygen species (ROS) generation and insufficient intracellular antioxidant activity. Table 2 shows mRNA level fold-changes calculated using 2^log FC^ and the DEG table of BMR Genomics of some mediators such as those of the glutathione peroxidase (GPXs) family, one of the important antioxidant enzymes and an important reactive oxygen species (ROS) free-radical scavenger in an organism. In particular, Glutathione peroxidase-7 (GPX7), a newly discovered non-selenium-containing protein with glutathione peroxidase activity, showed increased mRNA level expression (fold-change > 2.68) in HUVECs after 8 h of anoxia treatment. Mammalian GPX7 is a non-selenocysteine containing phospholipid hydroperoxide glutathione peroxidase activity that plays an important role in maintaining the redox state. When cells are exposed to oxidative stress, the unique pressure-sensor function of GPX7 can effectively detect redox levels and transmit ROS signals to redox-sensitive cells and thiol-containing proteins, further promoting protein folding and releasing non-targeted short interfering RNAs related to stress [44]. Previous studies have shown that the loss of GPx7 resulted in systemic oxidative stress damage, increased carcinogenesis, and shortened life span in mice [45,46,47,48,49].

In Table 2, there is also a clearly evident increase in the ribonucleoside-diphosphate reductase subunit M1 (*RRM1*, 2.1-fold) and ribonucleoside-diphosphate reductase subunit M2 (*RRM2*, 3.6-fold) that appears to be strongly involved in the cellular response to anoxia stimulus.

Ribonucleotide reductase (RR), comprising RRM1 and RRM2 subunits, is a unique enzyme that catalyzes the conversion of ribonucleotides (NDPs) to deoxyribonucleotides (dNDPs), which are the building blocks for DNA synthesis and thus essential for cell proliferation. This enzyme catalyzes a crucial step of de novo DNA synthesis by converting ribonucleoside diphosphates to deoxyribonucleoside diphosphates; tight control of the deoxyribonucleotide triphosphate (dNTP) pool is essential for cellular homeostasis [50]. Some papers have questioned the source of dNTPs in hypoxia and discussed probable mechanisms by which the RRM1/RRM2 enzymes are capable of retaining activity in hypoxia in order to preserve ongoing replication and avoid the accumulation of DNA damage [51]. Oxygen is an essential cofactor for mammalian ribonucleotide reductase to be capable of de novo synthesis of dNTPs and the upregulation of RRM1 and RRM2 found in our cell model is in accordance with the literature indicating that HUVEC cells are also able to sustain DNA replication in anoxic conditions. Regeneration of the ribonucleotide reductase is accomplished either by GSH-dependent glutaredoxin (Grx) or thioredoxin (Trx), which have been recognized as the primary electron donors to reduce RR [52]. Electrons for such a reduction come ultimately from NADPH via glutathione reductase (GR) and thioredoxin reductase (TR) [53]. Parallel studies demonstrated how GSH and Trx systems, which play a central role in the cellular redox state, are altered by O_2_ reduction [54] and are also involved in the control of reactive oxygen species (ROS), which will be investigated afterwards.

### 2.2. GSH Content in HUVECs after Anoxia

The exposure to severe hypoxic conditions results in the rapid usage of glutathione, leading to a marked alteration in the redox status of the intracellular milieu [55].

**Figure 5:**
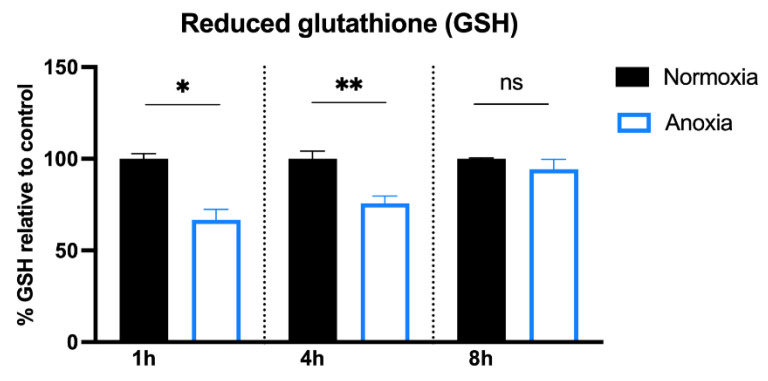
GSH level was determined via HPLC and normalized to protein content. GSH values were expressed as a percentage with respect to normoxic samples. Plotted values are the mean ± SD of three separate experiments. * *p* < 0.05, ** *p* < 0.005, ns: not significant.

Therefore, GSH levels in HUVEC were evaluated using HPLC at different time points (1, 4, and 8 h), as reported in [56]. GSH content decreased markedly after 1 h in anoxia and a similar profile was detected after 4 h. Only at 8 h were the cells able to restore their baseline levels, probably thanks to the activation of their antioxidant defense systems, as illustrated in Figure 5.

GSH could be restored from GSSG via GSR, or through its de novo synthesis (GCL, GSS), although expression of these enzymes seems to be unaffected by anoxic conditions (Table 2). However, due to the complexity of the system, this aspect of modulating GSH synthesis and degradation by anoxia requires further investigation. Importantly, the anoxia-dependent upregulation of the RR and the Gpx7 together with the drop in GSH after 1 h and 4 h presumes a shift to a more oxidized environment within the cells, suggesting that the direct application of redox-active molecules or overexpression of SIRT6 could represent a novel strategy to maintain redox balance and physiological redox signaling.

### 2.3. Reactome Functional Interaction Network

We considered it interesting to investigate, through the use of the Reactome Functional Interaction Network (https://reactome.org/PathwayBrowser/#TOOL=AT): URL (accessed on 21 June 2021) which possible pathways were implicated by these upregulated selected genes. The 50 most variable genes as well as ones such as *VEGFA*, whose increased fold-changes were calculated by RNA-Seq of the available BMR platform, were loaded onto the Reactome software tool for analysis of specific molecular pathways or sub-pathways. Supplementary Materials show how many of the genes we detected with an 8 h anoxia-related increase in expression were present in the 25 most relevant pathways sorted by *p*-value; for example, 3 of the detected genes were found to belong to the pool of genes involved in the following pathways: (1) cellular response to hypoxia, (2) signaling by VEGF, (3) platelet degranulation, and (4) response to elevated platelet cytosolic Ca^2+^ pathways.

### 2.4. Results on HUVEC-SIRT6 Transfected Cells after Anoxia Treatment

Biologic factors and processes influencing the behavior of hematopoietic cells during mobilization and transplantation have still not been fully discovered and understood; however, it is known that in the complex milieu of components participating in HSC maintenance, survival, proliferation, and differentiation [57,58], different elements such as adhesive molecules, growth factors, cytokines, proteases, miRNAs, and sirtuins are involved [59,60,61]. Among these factors, sirtuins may play an important role in the mobilization of HSCs through their deacetylating activity, which regulates many important processes related to the fate of HSCs, their metabolism, stress response, differentiation, aging, and apoptosis [62,63]. Some recent studies revealed a significant increase in the expression of sirtuins, in particular, SIRT6, that may be associated with the efficacy of hematopoietic stem cell mobilization [64]. SIRT6, whose activity depends on the availability of intracellular NAD+ [65], is involved in the regulation of a wide variety of processes, including metabolism, cancer, and inflammation. Among other SIRT family members, SIRT6 is unique, being endowed with deacetylase activity, but also with de-fatty acylase and mono-ADP-ribosyltransferase activities [66,67,68]. Moreover, it is also known that some sirtuin enzymes, which depend on NAD+ binding, can mediate the transcriptional activation of IL-8 and regulate hypoxia-inducible factor-1 α (HIF-1α) stabilization, which is routinely used to screen for hypoxic conditions [69,70].

HUVECs were engineered to overexpress SIRT6 with the aim to investigate the role of SIRT6 in the release of chemokines or interleukins in this anoxic cell model.

**Figure 6:**
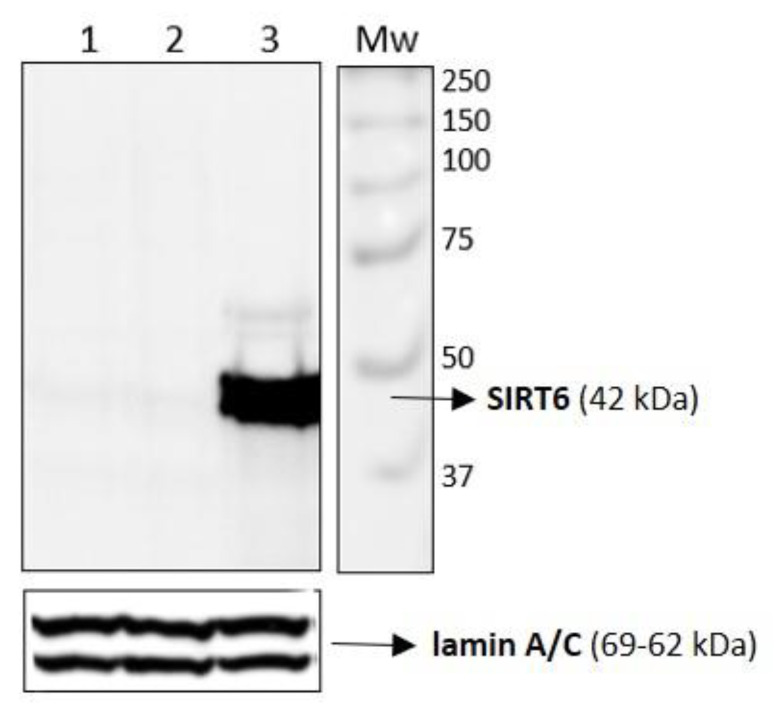
Western blotting analysis shows the SIRT6 protein band at 42kDa overexpressed in total cell extracts of a HUVEC-SIRT6wt sample (lane 3) in comparison to control native HUVEC (lane 1) and HUVEC-PBP (lane 2) samples.

Firstly, to evaluate the effective overexpression of SIRT6 in HUVEC-SIRT6wt samples, immunoblotting analysis of the protein was performed on cell extracts (Figure 6). It is evident that Sirtuin 6 protein (42 kDa) was remarkably overexpressed in HUVEC-SIRT6wt samples (lane 3, Figure 6) whereas in control cell samples (HUVEC wt, lane 1; HUVEC-PBP, lane 2), the band protein is undetectable, demonstrating the efficiency of the transfection.

**Figure 7:**
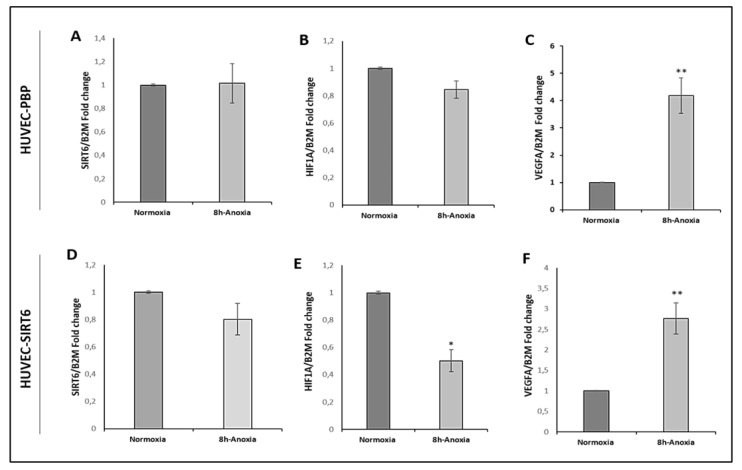
mRNA levels of *SIRT6*, *HIF1A* and *VEGFA* in cell extracts of anoxic HUVEC-PBP (**A**–**C**, respectively) and HUVEC-SIRT6 (**D**–**F**, respectively) samples versus the corresponding normoxic cell samples. * *p* < 0.05; ** *p* < 0.01.

*SIRT6* mRNA levels in cell extracts of native HUVEC, HUVEC-PBP and HUVEC-SIRT6wt samples were also analyzed both in normoxic and 8 h anoxic conditions (Figure 7).

The data showed that 8 h anoxia treatment significantly downregulates *HIF1A* mRNA levels (0.50 ± 0.08-fold change; *p* = 0.019) in HUVEC-SIRT6 cells (Figure 7E), whereas there was not a significant decrease (0.84 ± 0.06; *p* = 0.057) in HUVEC-PBP samples (Figure 7B). Moreover, anoxia exposure did not significantly alter *SIRT6* mRNA levels either in HUVEC-SIRT6 samples (0.80-fold change, Figure 7D) or in HUVEC-PBP samples (1.01-fold change, Figure 7A) in comparison with *SIRT6* mRNA levels of cells in normoxic conditions. The *VEGFA* mRNA levels in HUVEC-PBP and HUVEC-SIRT6 samples were also reported; after 8 h anoxia exposure, both cell samples presented a significant increase (3–4-fold change) in *VEGFA* expression levels (Figure 7C,F).

### 2.5. G6PDH, NADH and ROS Evaluation in HUVEC-SIRT6 Cells Exposed to Anoxic Conditions

**Figure 8:**
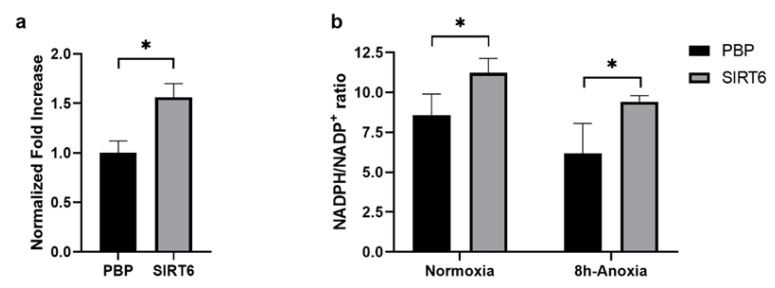
(**a**) G6PD activity in control (PBP) and SIRT6-overexpressing HUVEC cells in normoxic conditions. (**b**) NADPH/NADP^+^ ratio in PBP and SIRT6-overexpressing cells under normoxia (Ctrl) or exposed to 8 h anoxia treatment. * *p* < 0.05.

It was previously demonstrated that SIRT6 can prevent oxidative stress damage through upregulation of NADPH levels as a consequence of glucose-6-phosphate dehydrogenase (G6PD) activation. G6PD activity and NADPH levels were increased in SIRT6-overexpressing MCF-7 cells [71]. In agreement with these data, the overexpression of SIRT6 in HUVEC cells caused an increase in G6PD activity (HUVEC-SIRT6 versus HUVEC-PBP; Figure 8a) and in the NADPH/NADP+ ratio (Ctrl; Figure 8b). During the anoxia exposure (0.5% O_2_ for 8 h) of HUVEC-PBP samples (cells transduced with empty vector), the NADPH/NADP^+^ ratio decreased, consistent with NADPH utilization to reduce GSSG to GSH. The NADPH/NAD+ decline was significantly counteracted by SIRT6 overexpression (HUVEC-SIRT6 in 8 h anoxic conditions; Figure 8b).

**Figure 9:**
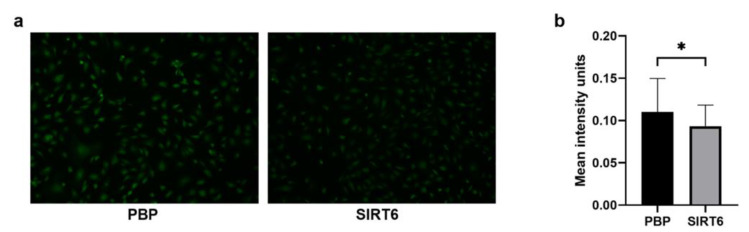
(**a**) ROS-positive HUVEC-SIRT6 and PBP cells after 8 h of anoxia (0.5% O_2_). Representative images acquired via GFP filter (Ex./Em. 470/525) with 10x magnification. (**b**) Fluorescence intensity quantitation of ROS-positive cells by CellProfiler Software. Statistical analysis was performed by using *n*= 610 cells, * *p* < 0.05.

It is also known that the role of the redox homeostasis in the maintenance of stem cell self-renewal and differentiation of stem cells plays a critical role [72,73]. Previous studies have implicated reactive oxygen species (ROS) and cytokines in the regulation of endothelial permeability. Some authors reported that prolonged hypoxia alters HUVEC permeability to increasing ROS generation, which amplifies cytokine production. IL-6 and IL-8 secretion increased 4-fold over 24 h in a pattern corresponding to changes in HUVEC permeability as measured by transendothelial electrical resistance [74]. In hypoxic conditions, production of ROS, as detected by a fluorescent probe, was significantly reduced in cells overexpressing SIRT6 (Figure 9), suggesting that the increased NADPH availability in SIRT6-overexpressing cells counteracts ROS production. 

### 2.6. Transcriptional Analysis of Cell Extracts from 8 h Anoxia-Treated HUVEC-SIRT6 Cells 

**Figure 10:**
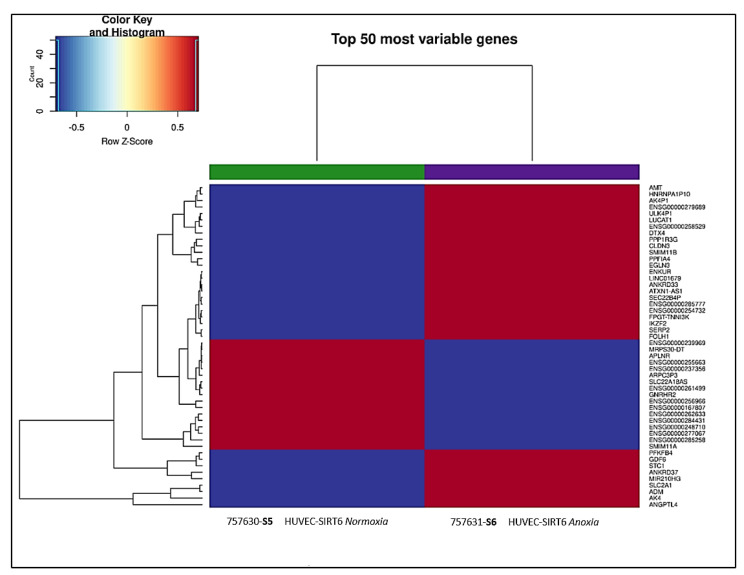
The 50 most variable genes involved in cellular responses differentially expressed in HUVEC−SIRT6 cells in anoxic (**S6**) versus normoxic (**S5**) conditions. Red color means upregulated genes; blue color means downregulated genes.

**Figure 11:**
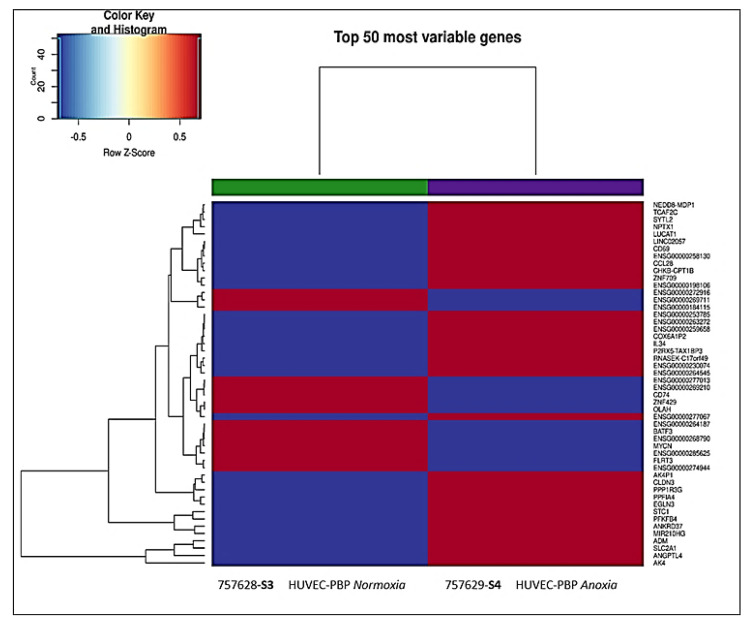
The 50 most variable genes involved in cellular responses differentially expressed in anoxic HUVEC−PBP (**S4**) versus HUVEC−PBP normoxic samples (**S3**). Red color means upregulated genes; blue color means downregulated genes.

The heatmap generated from transcriptional profiling BMR analysis performed using the total RNA derived from HUVEC-SIRT6 cells exposed for 8 h to anoxic conditions (*S6*) versus HUVEC-SIRT6 cells in normoxic conditions (*S5*) is reported in Figure 10, which shows the 50 most variable genes detected. Moreover, we calculated the mRNA fold-change (by using 2^log FC^ and DEG table of the available RNA-Seq BMR platform) of some other genes that, on the basis of the literature, could be involved in cell communication in the hematopoietic niche. We tried to understand which genes were effectively affected by the presence of SIRT6 and to relate this to results obtained with normoxic and anoxic HUVEC-PBP cells, for which the corresponding heatmap is reported in Figure 11.

**Figure 12:**
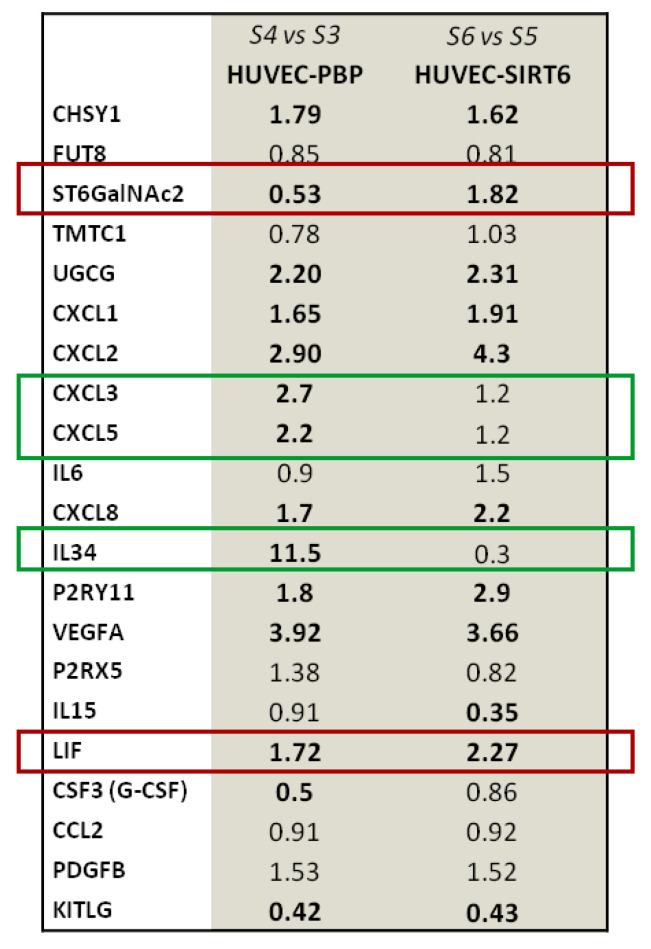
mRNA level expression of some of the up- (red boxes) or down- (green boxes) regulated genes by SIRT6 overexpression in HUVEC-SIRT6 sample compared to mRNA level expression in HUVEC-PBP sample after 8h anoxia (**S3**: normoxic HUVEC−PBP; **S4**: anoxic HUVEC−PBP; **S5**: normoxic HUVEC−SIRT6; **S6**: anoxic HUVEC−SIRT6).

Figure 12 shows the expression levels of some of the genes that were modulated during the 8 h anoxia treatment by the presence of overexpressed SIRT6 in HUVEC cells. For example, we found a downregulation of *CXCL3* (C-X-C motif chemokine ligand 3) and *CXCL5* (C-X-C motif chemokine ligand 5), with both showing a 1.2-fold change vs. a 2.7-and 2.2-fold change, respectively, in the HUVEC-PBP control samples in anoxic conditions. In particular, *IL-34* mRNA levels were remarkably downregulated in HUVEC-SIRT6wt cells (0.3-fold change) compared with the higher level found in control HUVEC-PBP samples (11.5-fold) in anoxic conditions. On the contrary, an upregulation of *ST6GALNAC2* (ST6 N-Acetylgalactosamide Alpha-2,6-Sialytransferase 2) was evident in HUVEC-SIRT6 cells (1.82-fold change vs. 0.53-fold in HUVEC-PBP control cells). Another target gene is the Leukemia inhibitory factor (*LIF*), which was upregulated (2.3-fold) in the presence of overexpressing SIRT6 in comparison with HUVEC-PBP control cells (1.72-fold) after 8 h of anoxia exposure. Some works describe LIF as a member of the interleukin-6 cytokine superfamily; it is a pleiotropic protein expressed in multiple types of tissues and cells which regulates an array of important biological functions. For example, LIF maintains the pluripotency of embryonic stem cells, while it induces the differentiation of several myeloid leukemia cells and inhibits their growth. LIF is frequently overexpressed in a variety of solid tumors including colorectal, breast, and skin cancers. However, the mechanism for LIF overexpression in tumors is not well-understood. Hypoxia is a hallmark of solid tumors, including colorectal cancers. Some authors have studied the effect of hypoxia on LIF expression in colorectal cancer cells. Data clearly demonstrated that hypoxia induces *LIF* expression, mainly through HIF-2α, and this is an important underlying mechanism for *LIF* overexpression in human colorectal cancers [75,76]. Using the Reactome software tool, which analyses specific molecular pathways or sub-pathways, we were able to identify in which of the 25 most relevant pathways the modulated genes in anoxic HUVEC-SIRT6 samples were present (Supplementary Materials). At least 3 genes were found in the pool of genes involved in “Cellular response to hypoxia” and “Signaling by VEGF pathways”.

**Figure 13:**
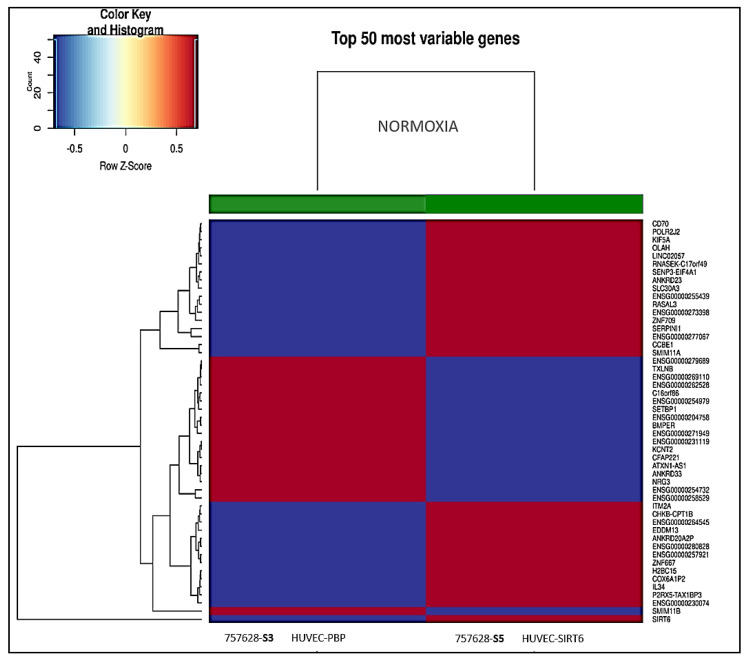
The 50 most variable genes found in HUVEC−PBP (**S3**) and HUVEC−SIRT6 (**S5**) samples when cells were maintained in normoxic conditions. Red color means upregulated genes; blue color means downregulated genes.

The transcriptional analyses performed with the RNA samples derived from HUVEC-PBP and HUVEC-SIRT6 cells in normoxic conditions are reported in Figure 13.

It is evident that the 50 most variable genes found are different from those detected in HUVEC-PBP and HUVEC-SIRT6 cells exposed to anoxic conditions. Notably, compared with control HUVEC-PBP samples in normoxic conditions, the genes modulated by the presence of an overexpressed SIRT6 in HUVEC-SIRT6 samples (HUVEC cells transfected with the SIRT6 cDNA construct) are involved in the regulation of pathways such as: (1) processes of DNA damage repair; (2) processing of DNA double strand break ends; (3) homology-directed repair (HDR); (4) HDR through homologous recombination or single-strand annealing; and (5) the Notch signaling pathway (Supplementary Materials). These indicate the role of the SIRT6 enzyme in the mechanisms of DNA damage repair in both physiological and oxidative stress conditions, in accordance with the literature. It is evident that these pathways are completely different from those specifically activated when HUVEC-SIRT6 samples are exposed to anoxic conditions (Supplementary Materials).

## 3. Materials and Methods

### 3.1. Cell Cultures 

HUVECs purchased from Lonza (Catalog #: C2517A; Human Umbilical Vein Endothelial Cells, Single Donor) were cultured in endothelial cell growth medium (EGM TM-2 BulletKit TM Medium, Lonza, Basel, Switzerland) in 25 cm^2^ flasks (Cell Star, Greiner bio-one, Kremsmünster, Austria) pre-coated with 25 µg/mL fibronectin (Sigma, St Louis, MO, USA) and maintained at 37 °C in a humidified 5% CO_2_ atmosphere. Cells were used at passages 4–8. Approximately 2.5 × 10^6^ cells were used for each experimental condition. HUVECs were exposed to anoxia using a Hypoxia Incubator Chamber (Chamber for generation of a hypoxic environment for tissue culture, StemCell Technologies, Vancouver, BC, Canada) flushed with the appropriate 95% N_2_ and 5% CO_2_ gas mixture for 15 min to reach anoxic conditions; normoxic cells were maintained in the incubator as control sample while the anoxic condition was maintained for all experimental time points reported in this work. For experiments involving HUVEC-pBABE-puro (PBP) and HUVEC-SIRT6 samples, cells were either engineered to stably overexpress Sirtuin 6 (SIRT6) or with the corresponding empty plasmid (PBP) by retroviral transduction as previously described. Positively infected HUVEC cells were selected with 1 μg/mL puromycin.

### 3.2. Western Blotting Analysis

Cells were lysed in buffer containing urea 6M, tiourea 2M, DTT 100 mM, Tris-HCl 30 mM, pH 7.5, triton 1%, and glycerol 9% supplemented with protease inhibitors (cComplete Mini; Roche, Basel, Switzerland); lysates were boiled for 7 min, sonicated twice at 100 Watt for 10 s and cleared by centrifugation at 16,000× *g* for 10 min, and the supernatants were recovered [2]. Appropriate amounts of proteins as determined by the Bio-Rad Protein Assay (Bio-Rad, Hercules, CA, USA) were resolved by 8% SDS polyacrylamide gel electrophoresis (SDS-PAGE) and afterward transferred onto nitrocellulose membranes (100 V, 70 min at 4 °C). The blots were probed with the following primary antibodies: anti-HIF-1α (#14179, monoclonal, recognizing amino acidic residues surrounding Lys460 that is codified by exon 10 of the *HIF1A* CDS1) and anti-SIRT6 (D8D12 Rabbit mAb #12486) from Cell Signaling Technology (Danvers, MA, USA); anti-β-actin (#VMA00048, monoclonal) from Bio-Rad (Hercules, CA, USA); and anti-Lamin A/C (#sc-376248, monoclonal) from Santa Cruz Biotechnology (Dallas, TX, USA). Immunoreactive bands were detected by horseradish peroxidase (HRP)-conjugated secondary antibodies (Bio-Rad, Hercules, CA, USA). Peroxidase activity was detected with the enhanced chemiluminescence detection method (WesternBright ECL, Advasta, Menlo Park, CA, USA) using the ChemiDoc MP Imaging System (Bio-Rad, Hercules, CA, USA). Quantification of the protein bands was performed using Image Lab analysis software version 5.2.1 (Bio-Rad, Hercules, CA, USA).

### 3.3. Real-Time Quantitative PCR

Gene-specific expression analyses were performed as already reported [2]. Fluorescence intensity of each amplified sample was measured with an ABI PRISM 7500 sequence detection system (Applied Biosystems, Foster City, CA, USA). All measurements were performed at least in triplicate and reported as the average value ± standard deviation of the mean (mean ± SD). Target gene values were normalized to *B2M* mRNA measurements, and expression data were calculated according to the 2-ΔΔCt method. Primers were designed using Primer 3 Plus, and their sequences are: VEGF-F: 5′-TCACAGGTACAGGGATGAGGACAC-3′; VEGF-R: 5′-CAAAGCACAGCAATGTCCTGAAG-3′. B2M-F: 5′-GCCTGCCGTGTGAACCAT-3′; B2M-R: 5′-CATCTTCAAACCTCCATGATGCT-3′; HIF1A-F: 5′-TCTG GGTTGAAACTCAAGCAACTG-3′; and HIF1A-R: 5′-CAACCGGTTTAAGGACACATTCTG-3′. SIRT6-F: 5′-CCTCCTCCGCTTCCTGGTC-3′; SIRT6-R: 5′-GTCTTACACTTGGCACATTCTTCC-3′.

### 3.4. mRNA-Seq Analysis

Transcriptomic analyses were performed by BMR Genomics S.r.l. (Via Redipuglia, 22, 35131 Padova, Italy) using 3 μg of total RNA obtained from 2 × 10^6^ control HUVECs or HUVECs maintained for 8 h in anoxic conditions. For total RNA isolation, cells were treated with the RNeasy Plus mini kit (Qiagen, Hilde, Germany) according to the manufacturer’s instructions as previously reported [2]. RNA-seq transcriptome analysis provided the heatmaps showing the 50 most variable genes found for normoxic and anoxic cell samples; these heatmaps allowed us to graphically identify those genes that are up- or downregulated after 8 h of anoxia treatment. In addition to this selected gene pool (the 50 most variable genes) found in normoxic or 8 h anoxia-treated native HUVEC samples (or HUVEC-PBP and HUVEC-SIRT6 samples), a differential gene expression analysis enabled us to research other genes of specific interest by using DEG Table (fold-changes for gene targets were calculated with the following formula: 2^log FC^).

### 3.5. Determination of Cytokine and Interleukin Release

Cell supernatants derived from HUVECs in normoxia and anoxia were collected at 8 h (cells used for RNA Seq), spun down at 1000× *g* for 10 min at 4 °C to remove any cell debris, and stored after addition of protease inhibitors (cComplete Mini; Roche, Basel, Switzerland) at −80 °C until analyses. A Bio-Plex Assay plate (Bio-Plex Pro Hu Screening Panel 27plx XPL) was purchased from Bio-Rad (Hercules, CA, USA) to detect human cytokines, interleukins, and other mediators and used according to the manufacturer’s recommendations. This kit permitted the detection of the following cytokines: FGF, G-CSF, IL-1β, IL-1α, IL-3, IL-16, IL-2, IL-5, IL-6, IL-8, LIF, IL-12, IL-15, MCP-1, SCF, SCGF-β, PDGF-BB, VEGF, MIF, M-CSF. Plates were read using a BioPlex^®^ 200 instrument (Bio-Rad, Hercules, CA, USA), and interleukin concentrations (expressed as picograms per milliliter) were calculated by use of a standard curve and software provided by the manufacturer (Bio-Plex manager software, v.6.1) and normalized by protein content defined by the Bradford assay.

### 3.6. NADP(H) Evaluation

HUVEC cells were plated at a density of 2 × 10^5^ cells/well in 12-well plates, cultured for 8 h in normoxic or anoxic conditions, harvested, and lysed in 0.1 mL 0.6 M perchloric acid (PCA) (for NADP+) or 0.1 M NaOH (for NADPH) at 4 °C. Cell extracts were centrifuged for 3 min at 16,000× *g*, and the supernatants were collected and neutralized: samples in PCA were neutralized by diluting the extracts in 100 mM sodium phosphate buffer (pH 8), whereas samples in NaOH were warmed at 72 °C for 10 min and neutralized in 10 mM Tris-HCl, pH 6. The intracellular NADP(H) content was assessed with a sensitive enzyme cyclic assay, which exploits the use of glucose-6-phosphate dehydrogenase (G6PD): 0.1 mL of the cycling reagent [10 mM glucose-6-phosphate, 0.02 U/mL G6PD, 20 μM resazurin, 5 μg/mL diaphorase, 10 μM FMN, 10 mM nicotinamide and 100 mM sodium phosphate, pH 8.0] were added to each well containing 0.1 mL of the diluted samples. The increase in resorufin fluorescence (544 nm excitation, 590 nm emission) was measured every minute over a 4 h period using a fluorescence plate reader (Fluostar Optima, BMG Labtechnologies GmbH, Offenburg, Germany). A NADP(H) standard curve was always run in parallel in each assay. NADP(H) levels in each sample were normalized to the protein content as determined by the Bradford assay.

### 3.7. Assay of Glucose-6-Phosphate Dehydrogenase Activity

HUVECs were lysed in ice-cold buffer [25 mM Tris-HCl (pH 7.4), 1 mM EDTA, and protease inhibitors] by brief sonication. The lysates were centrifuged at 10,000× *g* for 10 min at 4 °C and the supernatants (100 μg protein) were assayed for glucose-6-phosphate dehydrogenase (G6PD) activity by measuring the reduction of NADP^+^ in the reaction buffer [100 mM Tris-HCl, pH 7.4, 0.5 mM EDTA, 10 mM MgCl2, 0.2 mM NADP^+^, and 0.6 mM glucose-6-phosphate] at 25 °C. NADPH production was monitored at 355 nm excitation, 460 nm emission, using a fluorescence plate reader (see above).

### 3.8. ROS Assay

The ROS-ID^®^ Hypoxia/Oxidative stress detection kit (Enzo Life Sciences, Farmingdale, NY, USA) was used according to the manufacturer’s protocols to evaluate the total ROS production induced in anoxic cells. HUVECs were seeded at a density of 0.01 × 10^4^ cells/well in a 96-well plate. Cells were incubated for 8 h in a Clariostar plus microplate reader (BMG Labtech, Ortenberg, Germany). The level of ROS was determined with the microscope Evos M5000 (Thermo Fisher Scientific, Waltham, MA, USA) via a GFP filter (Ex./Em. 470/525), whereas anoxic cells were acquired via the Texas red filter (Ex./Em. 585/628).

### 3.9. Glutathione Detection

HUVECs (0.5 × 10^6^ cells/flask) were washed twice with PBS and lysed with 100 µL of lysis buffer (0.1% Triton X-100, 0.1 M Na_2_HPO_4_, 5 mM EDTA, pH 7.5) followed by 15 µL of 0.1 N HCl and 140 µL of precipitating solution (100 mL containing 1.67 g (*w/v*) of glacial metaphosphoric acid, 0.2 g (*w/v*) of disodium EDTA and 30 g (*w/v*) of NaCl). After centrifugation at 12,000× *g* for 10 min at 4 °C, pellets were resuspended in 100 µL 0.1 N NaOH for protein quantification via the Bradford assay (Bio-Rad, Hercules, CA, USA). Supernatants were collected and 25% (*v/v*) Na_2_HPO_4_ 0.3 M and 10% (*v/v*) DTNB were added for thiol determination by HPLC. A 5 µm, 150 × 4.6 mm BDS Hypersil^TM^ C18 column (Thermo Scientific, Waltham, MA, USA) was used for these studies. The mobile phase consisted of 10 mM KH_2_PO_4_ solution, pH 6.0 (buffer A), and buffer A containing 60% (*v/v*) of acetonitrile (buffer B). All solutions were filtered through a 0.22 µm Millipore filter. The elution conditions were: 10 min 100% buffer A, and gradually up to 100% buffer B for 20 min. Buffer B was hold for 5 min before returning the gradient to 100% buffer A within 3 min. The initial conditions were restored in 4 min. The flow rate was 1 mL/min and detection was carried out at 330 nm. Analyses were performed at room temperature and quantitative measurements were obtained by injection of known concentration standards and normalized protein content.

### 3.10. Statistical Analysis

Statistics and graphical representations were performed using GraphPad Prism^TM^ 9 (Boston, MA, USA). Student’s *t*-test and ANOVA test performed with Past Software version 3 were used for statistical analysis of the data; differences between groups were considered statistically significant when *p* < 0.05. Quantitative analyses of the images were accomplished with CellProfiler Software.

## 4. Conclusions

Depending on the oxygen content in the stem cell microenvironment, a number of adaptive physiological responses occur that regulate metabolism, redox homeostasis and vascular remodeling. Hypoxia has been linked to stem cell quiescence, whereas normoxia is thought to be required for stem cell activation. Given the importance of understanding stem cell biology, the effect of microenvironment and oxygen tension is of interest. However, the regulation of the stem cell microenvironment by normoxic/hypoxic conditions has yet to be defined, and there are few works that report observations on the effect of anoxia in vitro on bone marrow cultures. 

We found that marked anoxia exposure of endothelial cells triggers the transcription of a large number of genes involved in a variety of cellular processes such as glycolysis, angiogenesis, and cell proliferation, similar to those studied in hypoxic conditions, that are aimed at minimizing the deleterious effects caused by oxygen insufficiency at the cellular level. In recent years, the sirtuin family (SIRT1—SIRT7) has emerged as key regulators of many important biological processes, depending on their enzymatic activity, subcellular localization, and target specificity [77,78]. Among the seven sirtuins, SIRT6 is a chromatin-binding protein with diverse roles in genome stability, glucose metabolism, tumor suppression, and the organismal lifespan [71]. Previous studies have shown that SIRT6 deacetylates H3K9ac or H3K56ac and acts as a transcriptional co-repressor of the NF-kB-, C-JUN-, MYC-, and HIF-1α-mediated pathways in a tissue- and context-dependent manner. Many of these pathways have been implicated in the regulation of adult stem cell function and maintenance. However, there is no clear evidence indicating whether SIRT6 is functional relevant to HSC biology. Interestingly, the *CXCL3*, *CXCL5*, and *IL-34* genes, whose mRNA levels are upregulated in native HUVECs after anoxia exposure, become downregulated by SIRT6 overexpression in HUVEC-SIRT6 cells exposed to anoxia for the same amount of time. Moreover, while *LIF* mRNA expression was not significantly affected by 8 h anoxia exposure in native HUVECs, it becomes upregulated in the presence of SIRT6. Therefore, the ability of Sirtuin 6 to modulate specific genes is evident.

It is known that *CXCL5* encodes a protein that is a member of the CXC subfamily of chemokines which recruit and activate leukocytes and which are classified by function (inflammatory or homeostatic) or by structure. This protein is proposed to bind the G-protein-coupled receptor chemokine (C-X-C motif) receptor 2 to recruit neutrophils, to promote angiogenesis, and to remodel connective tissues. It is widely identified in different cells and organs, such as endothelial cells and brain [79]. This protein is thought to play a role in cancer cell proliferation, migration, and invasion. A recent paper [80] reports CXCL5 (that binds with CXCR1 and CXCR2 and activates the p38 MAP kinase signaling pathways) to be upregulated in ischemic stroke. The authors constructed an ischemic/hypoxic model in human brain microvascular endothelial cells (BMECs) and investigated the function of CXCL5 and its potential value as a therapeutic target for ischemia-induced brain disease. The results demonstrated that *CXCL5* silencing attenuated the ischemic/hypoxic-induced injury in human BMECs. Furthermore, the p38 inhibitor SB203580 significantly abolished the function of CXCL5 in model cells. In our anoxic cell model, the presence of overexpressed Sirtuin 6 is able to downregulate the expression of *CXCL5* which, in contrast, is upregulated in native HUVECs exposed for 8 h to anoxia conditions. Some authors reported a chemotaxis assay showing that CXCL5 induces the migration of hematopoietic stem cells, suggesting that the differential regulation of the chemokine CXCL5 between the endosteal osteoblast (OB) niche and endothelial cells is involved in HSC mobilization from the OB niche or bone marrow to peripheral blood [81].

*ST6GalNac2*, known to have a role in cancer, [82] also appears to be upregulated in the presence of overexpressed SIRT6 in HUVEC-SIRT6 samples.

Concerning LIF, it has already been reported that it plays an important role in a wide array of biological processes, including stem cell self-renewal. Hypoxia plays a critical role in *LIF* overexpression in solid tumors. The expression of *LIF* is also regulated by many cytokines. In cultured normal human bone marrow stromal cells, IL-1α, IL-1β, TGF-β, and TNF-α can all increase the transcription of LIF mRNA [83]. In our anoxic model, SIRT6 induces an upregulation of this factor, which could in turn lead to the modulation of interleukins and chemokines.

We have shown here that SIRT6 is able to maintain the NADPH/NADP^+^ ratio in an anoxic environment. Sirtuins are indispensable for the maintenance of cellular redox homeostasis as they serve as key regulators of oxidative stress mechanisms. Sirtuins can both directly and indirectly regulate a wide variety of targets associated with primary antioxidant responses, including redox metabolic enzymes (such as G6PD), DNA repair enzymes, transcription factors, and co-factors [84]. This result is in line with the previously reported role for SIRT6 in preventing oxidative stress damage induced by H_2_O_2_ or by doxorubicin in breast cancer cells; this occurs through upregulation of NADPH levels as a consequence of G6PD activation [71]. In addition to this mechanism, in human mesenchymal cells, SIRT6 can prevent oxidative stress by transactivating NRF2-regulated antioxidant genes, including heme oxygenase 1 [85]. Finally, the increased levels of NADPH observed in SIRT6-overexpressing cells is in line with a recent report demonstrating that SIRT6 inhibits NADPH oxidase expression and activity in endothelial cells [86].

Of note, our results show that, in normoxic conditions, the stable transfection of endothelial HUVEC cells with the construct for the overexpression of Sirtuin 6 induces a pool of genes involved in the activation of molecular pathways modulated by Notch. The evolutionarily conserved “Notch signaling pathway” functions as a major mediator of cell fate determination during development and regulates several cellular functions including differentiation, proliferation, cell survival, and hematopoiesis process [87]. Interestingly, our results show that after 8 h of anoxia treatment of HUVEC-SIRT6 cells, there was a significant increase in VEGFA mRNA levels as well as activation of the molecular pathways categorized as “Cellular response to hypoxia” and “Signaling by VEGF”, indicating the induction of those molecular mechanisms that can support the expansion of the hematopoietic stem cell population.

## Figures and Tables

**Table 1:** Interleukin and chemokine protein secretion in the medium of HUVEC cells incubated in normoxia (**S1**) or 8 h anoxia (**S2**) was determined by multiplex suspension and Immunomagnetic assays (BioPlex, BioRad, Hercules, CA, USA). Values are expressed as picograms per milligram of total proteins and compared as fold-change (**S2** versus **S1**).

Samples	IL-6	IL-8	MCP-1	IL-1b	IL-2	IL-5	PDGF-BB	IP-10
S1	3167	110,236	8097	43	102	196	832	1222
S2	23,941	358,257	40,761	34	122	444	716	63,475
**S2** vs. **S1** (fold)	**7.56**	**3.25**	**5.03**	**0.79**	**1.19**	**2.26**	**0.86**	**51.96**

**Table 2:** Evaluation of mRNA levels of some genes involved in glutathione metabolism.

Gene Description	Fold Change
GSR, gluthatione reductase (NADPH)	1.40
IDHI, isocitrate dehydrogenase	1.27
PGD, 6-phosphogluconate dehydrogenase	1.01
G6PD, glucose-6 phosphate deydrogenase	0.74
GPX1, glutathione peroxidase 1	1.31
GPX1P1, glutathione peroxidase, pseudogene 1	1.34
GPX3, glutathione peroxidase 3	0.76
GPX4, glutathione peroxidase 4	1.21
**GPX7, glutathione peroxidase 7**	2.68
GPX8, glutathione peroxidase 8	1.44
**RRM1, ribonucleoside-diphosphate reductase subunit M1**	2.10
**RRM2, ribonucleoside-diphosphate reductase subunit M2**	3.56
OPLAH, 5 oxoprolinase (ATP-hydrolysing)	0.71
GCLM, glutamate-cysteine ligase regulatory subunit	0.91
GCLC, glutamate-cisteine ligase (6.3.2.2)	0.99
GSS, glutathione synthase (6.3.2.3)	1.06
SLC6A9, glycin transporter (Glyt 1)	0.70
SLC1A1, glutamate transporter	1.51
SLC7A11, cystine glutamate transporter	1.40

## Supplementary Materials

The following supporting information can be downloaded at: https://www.mdpi.com/article/10.3390/ijms24065157/s1.
