# Supplementary material for: Anoxia Rapidly Induces Changes in Expression of a Large and Diverse Set of Genes in Endothelial Cells
Source: Int J Mol Sci. 2023 Mar 8;24(6):5157. doi: 10.3390/ijms24065157 (PMC10049254; doi:10.3390/ijms24065157)

Supplementary Material

The 50 most variable genes (plus VEGFA gene) obtained by BMR transcriptome analysis were used as query in the Reactome software tool to analyze a database of cellular molecular pathways where genes modulated in anoxic native HUVEC (*S2*) (**Figure S1**), anoxic HUVEC-SIRT6 (*S6*) (**Figure S2**) versus the respective control samples, and normoxic HUVEC-SIRT6 (*S5*) respect to normoxic HUVEC-PBP (*S3*) sample (**Figure S3**) are involved. The tables show the 25 most relevant pathways to which the selected 51 genes belong. In each figure, only the pathways where at least 3 genes are involved have been indicated.

Figure S1. Reactome software tool analysis of specific molecular pathways or sub-pathways where some genes of **8h-anoxia** treated native (*S2*) HUVEC samples (anoxic versus (*S1*) **normoxic** HUVEC sample) were found among the 25 most relevant pathways. At least 3 genes were found in pool genes involved in specific pathways (indicated by the arrows).

The following table shows the 25 most relevant pathways sorted by p-value.

Pathway name	Entities				Reactions	
	found	ratio	p-value	FDR*	found	ratio
VEGF ligand-receptor interactions	2 / 8	5.50e-04	4.90e-04	0.034	3 / 4	2.96e-04
VEGF binds to VEGFR leading to receptor dimerization	2 / 8	5.50e-04	4.90e-04	0.034	2 / 3	2.22e-04
Regulation of gene expression by Hypoxia-inducible Factor	2 / 15	0.001	0.002	0.078	1 / 7	5.18e-04
TFAP2 (AP-2) family regulates transcription of growth factors and their receptors	2 / 21	0.001	0.003	0.111	2 / 18	0.001
Cellular response to hypoxia	3 / 86	0.006	0.005	0.135	10 / 20	0.001
Defective SLC2A1 causes GLUT1 deficiency syndrome 1 (GLUT1DS1)	1 / 2	1.38e-04	0.008	0.157	1 / 1	7.40e-05
ATF4 activates genes in response to endoplasmic reticulum stress	2 / 34	0.002	0.008	0.157	1 / 7	5.18e-04
PERK regulates gene expression	2 / 42	0.003	0.012	0.204	1 / 11	8.14e-04
FOXO-mediated transcription of oxidative stress, metabolic and neuronal genes	2 / 49	0.003	0.017	0.204	2 / 34	0.003
Signaling by VEGF	3 / 137	0.009	0.018	0.204	49 / 86	0.006
Platelet degranulation	3 / 139	0.01	0.018	0.204	1 / 11	8.14e-04
Transcriptional regulation by the AP-2 (TFAP2) family of transcription factors	2 / 52	0.004	0.019	0.204	2 / 44	0.003
Response to elevated platelet cytosolic Ca2+	3 / 146	0.01	0.021	0.207	1 / 14	0.001
Toxicity of botulinum toxin type E (botE)	1 / 8	5.50e-04	0.031	0.209	3 / 5	3.70e-04
Lactose synthesis	1 / 8	5.50e-04	0.031	0.209	1 / 3	2.22e-04
Release of apoptotic factors from the mitochondria	1 / 8	5.50e-04	0.031	0.209	1 / 4	2.96e-04
SMAC, XIAP-regulated apoptotic response	1 / 8	5.50e-04	0.031	0.209	1 / 10	7.40e-04
O-linked glycosylation of mucins	2 / 73	0.005	0.035	0.209	1 / 17	0.001
Defective CHST14 causes EDS, musculocontractural type	1 / 9	6.19e-04	0.035	0.209	1 / 1	7.40e-05
Defective CHST3 causes SEDCJD	1 / 9	6.19e-04	0.035	0.209	1 / 1	7.40e-05
Toxicity of botulinum toxin type D (botD)	1 / 9	6.19e-04	0.035	0.209	3 / 5	3.70e-04
Toxicity of botulinum toxin type F (botF)	1 / 9	6.19e-04	0.035	0.209	3 / 5	3.70e-04
Defective CHSY1 causes TPBS	1 / 10	6.88e-04	0.039	0.209	2 / 2	1.48e-04
Calcitonin-like ligand receptors	1 / 11	7.56e-04	0.043	0.209	1 / 4	2.96e-04
Ficolins bind to repetitive carbohydrate structures on the target cell surface	1 / 12	8.25e-04	0.047	0.209	3 / 3	2.22e-04

Figure S2 Reactome software tool analysis of specific molecular pathways or sub-pathways where some genes of **8h-anoxia** treated (*S6*) HUVEC-**SIRT6** (versus normoxic (*S5)* HUVEC-**SIRT6** sample) were found among the 25 most relevant pathways. At least 3 genes were found in pool genes involved in specific pathways (indicated by the arrows).

The following table shows the 25 most relevant pathways sorted by p-value.

Pathway name	Entities				Reactions	
	found	ratio	p-value	FDR*	found	ratio
VEGF ligand-receptor interactions	2 / 8	5.50e-04	4.73e-04	0.024	3 / 4	2.96e-04
VEGF binds to VEGFR leading to receptor dimerization	2 / 8	5.50e-04	4.73e-04	0.024	2 / 3	2.22e-04
Hormone ligand-binding receptors	2 / 13	8.94e-04	0.001	0.041	2 / 5	3.70e-04
Regulation of gene expression by Hypoxia-inducible Factor	2 / 15	0.001	0.002	0.041	1 / 7	5.18e-04
Regulation of glycolysis by fructose 2,6-bisphosphate metabolism	2 / 18	0.001	0.002	0.047	3 / 4	2.96e-04
TFAP2 (AP-2) family regulates transcription of growth factors and their receptors	2 / 21	0.001	0.003	0.051	2 / 18	0.001
Cellular response to hypoxia	3 / 86	0.006	0.005	0.067	10 / 20	0.001
Defective SLC2A1 causes GLUT1 deficiency syndrome 1 (GLUT1DS1)	1 / 2	1.38e-04	0.008	0.094	1 / 1	7.40e-05
Signaling by VEGF	3 / 137	0.009	0.017	0.179	49 / 86	0.006
Transcriptional regulation by the AP-2 (TFAP2) family of transcription factors	2 / 52	0.004	0.018	0.179	2 / 44	0.003
PKA-mediated phosphorylation of key metabolic factors	1 / 7	4.81e-04	0.027	0.216	1 / 5	3.70e-04
IRF3 mediated activation of type 1 IFN	1 / 7	4.81e-04	0.027	0.216	1 / 6	4.44e-04
Lactose synthesis	1 / 8	5.50e-04	0.031	0.216	1 / 3	2.22e-04
PP2A-mediated dephosphorylation of key metabolic factors	1 / 9	6.19e-04	0.035	0.222	1 / 4	2.96e-04
Calcitonin-like ligand receptors	1 / 11	7.56e-04	0.042	0.222	1 / 4	2.96e-04
Glycine degradation	1 / 15	0.001	0.057	0.222	1 / 3	2.22e-04
Transcriptional regulation of white adipocyte differentiation	2 / 109	0.007	0.068	0.222	1 / 18	0.001
Glycolysis	2 / 110	0.008	0.069	0.222	3 / 24	0.002
IRF3-mediated induction of type I IFN	1 / 19	0.001	0.072	0.222	1 / 5	3.70e-04
Receptor-type tyrosine-protein phosphatases	1 / 20	0.001	0.075	0.222	1 / 6	4.44e-04
Regulation of innate immune responses to cytosolic DNA	1 / 21	0.001	0.079	0.222	4 / 9	6.66e-04
Serotonin Neurotransmitter Release Cycle	1 / 23	0.002	0.086	0.222	2 / 4	2.96e-04
ZBP1(DAI) mediated induction of type I IFNs	1 / 23	0.002	0.086	0.222	1 / 11	8.14e-04
STING mediated induction of host immune responses	1 / 24	0.002	0.09	0.222	1 / 19	0.001
Acetylcholine Neurotransmitter Release Cycle	1 / 26	0.002	0.097	0.222	2 / 6	4.44e-04

Figure S3 Reactome software tool analysis of specific molecular pathways or sub-pathways where some genes of **normoxic** (*S5*) HUVEC-**SIRT6** (versus normoxic (*S3*) HUVEC-**PBP** sample) were found among the 25 most relevant pathways. At least 3 genes were found in pool genes involved in specific pathways (indicated by the arrows).

The following table shows the 25 most relevant pathways sorted by p-value.

Pathway name	Entities				Reactions	
	found	ratio	p-value	FDR*	found	ratio
Pre-NOTCH Transcription and Translation	3 / 62	0.005	0.003	0.091	3 / 28	0.002
Pre-NOTCH Expression and Processing	3 / 78	0.007	0.005	0.091	3 / 38	0.003
Processing of DNA double-strand break ends	3 / 81	0.007	0.006	0.091	3 / 17	0.001
RNA Polymerase I Promoter Opening	2 / 32	0.003	0.009	0.091	1 / 2	1.48e-04
Packaging Of Telomere Ends	2 / 33	0.003	0.009	0.091	2 / 2	1.48e-04
DNA methylation	2 / 34	0.003	0.01	0.091	7 / 7	5.18e-04
Activated PKN1 stimulates transcription of AR (androgen receptor) regulated genes KLK2 and KLK3	2 / 36	0.003	0.011	0.091	8 / 11	8.14e-04
SIRT1 negatively regulates rRNA expression	2 / 37	0.003	0.012	0.091	3 / 5	3.70e-04
Cleavage of the damaged purine	2 / 38	0.003	0.012	0.091	2 / 9	6.66e-04
Depurination	2 / 39	0.003	0.013	0.091	4 / 19	0.001
Recognition and association of DNA glycosylase with site containing an affected purine	2 / 39	0.003	0.013	0.091	2 / 10	7.40e-04
HDR through Homologous Recombination (HRR) or Single Strand Annealing (SSA)	3 / 114	0.01	0.014	0.091	3 / 42	0.003
PRC2 methylates histones and DNA	2 / 42	0.004	0.015	0.091	4 / 4	2.96e-04
Defective pyroptosis	2 / 42	0.004	0.015	0.091	1 / 3	2.22e-04
Cleavage of the damaged pyrimidine	2 / 42	0.004	0.015	0.091	1 / 20	0.001
Depyrimidination	2 / 42	0.004	0.015	0.091	2 / 41	0.003
Recognition and association of DNA glycosylase with site containing an affected pyrimidine	2 / 42	0.004	0.015	0.091	1 / 21	0.002
Homology Directed Repair	3 / 120	0.01	0.016	0.091	3 / 50	0.004
Condensation of Prophase Chromosomes	2 / 45	0.004	0.017	0.091	8 / 10	7.40e-04
ERCC6 (CSB) and EHMT2 (G9a) positively regulate rRNA expression	2 / 45	0.004	0.017	0.091	2 / 4	2.96e-04
Base-Excision Repair, AP Site Formation	2 / 46	0.004	0.018	0.091	6 / 62	0.005
Inhibition of DNA recombination at telomere	2 / 48	0.004	0.019	0.091	3 / 4	2.96e-04
Nonhomologous End-Joining (NHEJ)	2 / 52	0.004	0.022	0.091	4 / 13	9.62e-04
Deposition of new CENPA-containing nucleosomes at the centromere	2 / 54	0.005	0.024	0.091	2 / 4	2.96e-04
Nucleosome assembly	2 / 54	0.005	0.024	0.091	2 / 4	2.96e-04

Figure S4. Uncropped images of western blot results shown in **Figure 2c** and in **Figure 8**.

In each image, the *lanes* that have been used for the final version of Figure 2c and the final version of Figure 8 have been indicated with the symbol §.

Uncropped Figure 2c

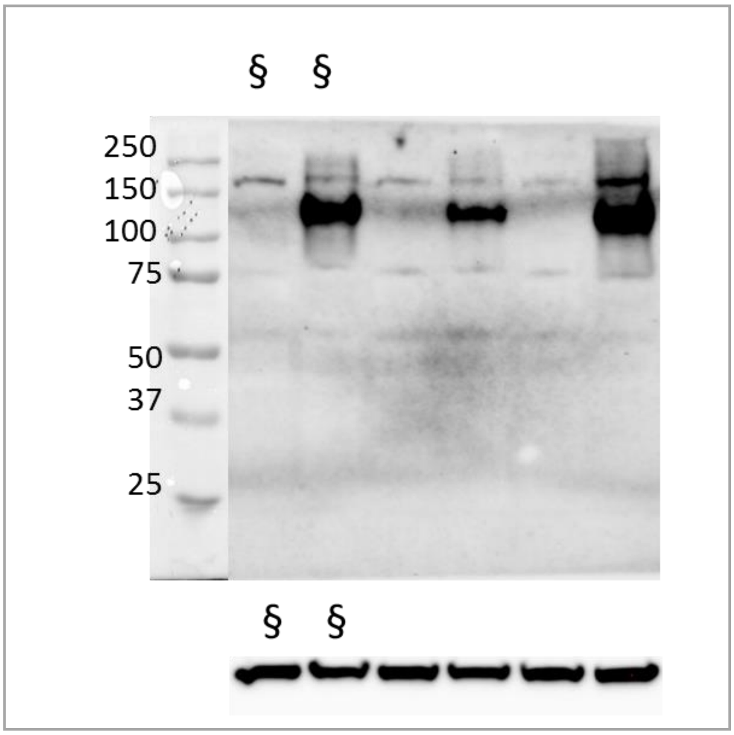

Uncropped Figure 8

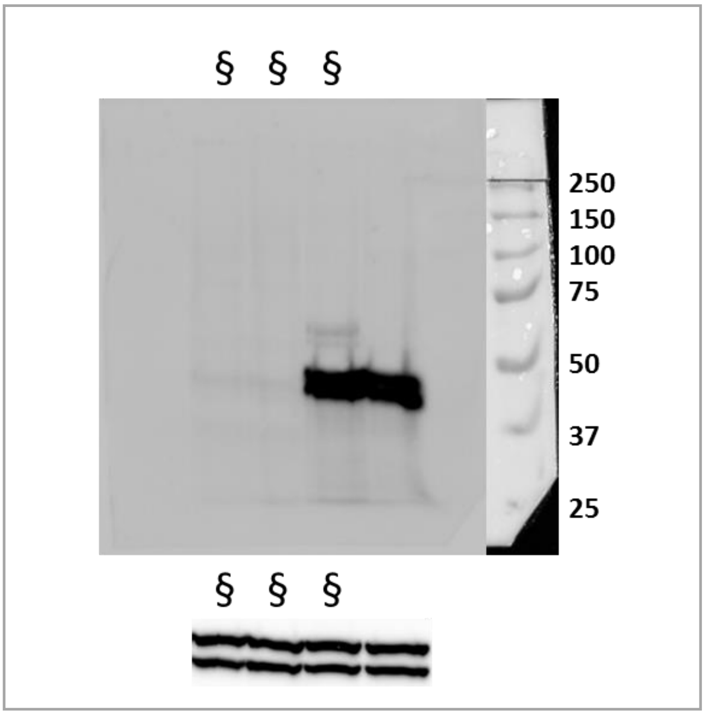

Supplement: Supplementary file 1 [file ijms-24-05157-s001.zip › ijms-2239713-supplementary.pdf]
